# Strain Monitoring and Numerical Simulation Analysis of Nuclear Containment Structure During Containment Tests

**DOI:** 10.3390/s25165197

**Published:** 2025-08-21

**Authors:** Xunqiang Yin, Weilong Yang, Junkai Zhang, Min Zhao, Jianbo Li

**Affiliations:** 1 College of Civil Engineering and Architecture, Dalian University, Dalian 116622, China; 2 Department of Civil and Hydraulic Engineering, Dalian University of Technology, Dalian 116024, China

**Keywords:** strain monitoring, containment test, surface-mounted strain sensors, numerical simulation, nuclear containment

## Abstract

Strain monitoring during the service life of a nuclear containment structure is an effective means to evaluate whether the structure is operating safely. Due to the failure of embedded strain sensors, surface-mounted strain sensors should be installed on the outer wall of the structure. However, whether the data from these substitute sensors can reasonably reflect the internal deformation behavior requires further investigation. To ensure the feasibility of the added strain sensors, a refined 3D model of a Chinese Pressurized Reactor (CPR1000) nuclear containment structure was developed in ANSYS 19.1 to study the internal and external deformation laws during a containment test (CTT). Solid reinforcement and cooling methods were employed to simulate prestressed cables and pre-tension application. The influence of ordinary steel bars in concrete was modeled using the smeared model, while interactions between the steel liner and concrete were simulated through coupled nodes. The model’s validity was verified against embedded strain sensor data recorded during a CTT. Furthermore, concrete and prestressed material parameters were refined through a sensitivity analysis. Finally, the variation law between the internal and external deformation of the containment structure was investigated under typical CTT loading conditions. Strain values in the wall thickness direction exhibited an essentially linear relationship. Near the equipment hatch, however, the strain distribution pattern was significantly influenced by the spatial arrangement of prestressed cables. Refined FEM and sensor systems are vital containment monitoring tools. Critically, surface-mounted strain sensors offer a feasible approach for inferring internal stress states and deformation behavior. This study provides theoretical support and a technical foundation for the safe assessment and maintenance of nuclear containment structures during operational service.

## 1. Introduction

Nuclear energy, recognized as a crucial clean resource for optimizing the energy structure, offers distinct advantages in addressing energy demand, mitigating environmental pollution, and combating climate change [1,2,3]. However, unlike other renewable energy sources, there are potential radiation safety issues with nuclear energy utilization. Should a severe accident occur at a nuclear power plant, leading to the large-scale release of radionuclides, the consequences would be catastrophic. Therefore, safety must be given top priority during nuclear energy utilization [4].

As the third safety barrier for nuclear power plants (NPPs), the containment structure must possess sufficient strength and sealing performance to contain the large amounts of radioactivity, high temperatures, and the high-pressure water mixture released by the Loss of Coolant Accident (LOCA) in the reactor. This ensures that the workers and the surrounding environment are safe. According to RCC-G86 [5], containment structures must undergo performance tests after the completion of construction, the initial refueling overhaul, and every 10 years of commercial operation. This is called a containment test (CTT), which assesses whether the strength and sealing performance of the containment structure meet the requirements under simulated LOCA conditions to ensure nuclear safety functions. Therefore, assessing the strength and sealing performance of the containment structure is vital for NPPs to maintain safety in the event of accidents and to estimate the lifespan of the structure. Strain monitoring during the service life of a nuclear containment structure is an effective means to evaluate whether it is operating safely [6,7,8,9,10].

**Figure 1:**
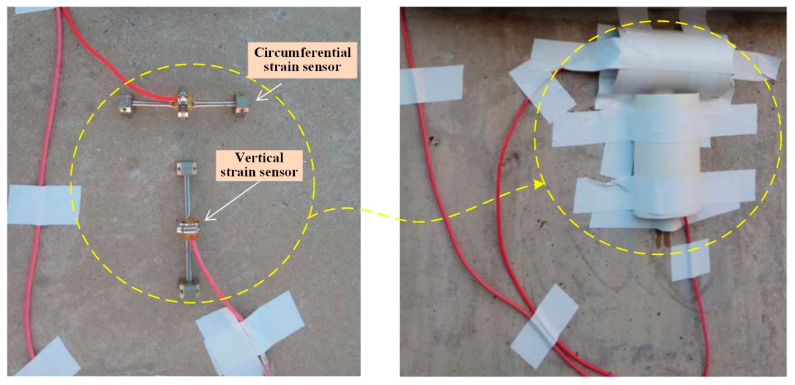
Installation and protection of surface-mounted strain sensors.

The Enceinte Étanche Instrumentation (EAU) system is the core instrumentation and monitoring system in the containment structure of a CPR1000 nuclear power unit. It provides real-time monitoring of the containment’s structural integrity and internal environmental parameters to ensure its leak-tightness and safety under both normal operation and accident conditions. The EAU primarily consists of the following components embedded within the containment concrete: a plumb line measurement system, a strain and temperature measurement system, a vertical tendon prestress measurement system, a water level tank measurement system, and a convergence meter system. Within the strain and temperature measurement system, strain and thermocouple temperature sensors are typically embedded within the containment structure during construction to monitor strain and temperature under operational internal pressure loads in real time. This system provides critical technical data for evaluating the safety status of the containment structure. However, various factors can lead to embedded strain sensors becoming partially damaged or to deviations from their original design positions during construction. This can result in either unreadable data or data that fails to accurately reflect the design requirements. Furthermore, certain damaged embedded sensors are irreparable or irreplaceable yet monitoring data from their locations remains indispensable. Consequently, it is critically important to develop alternative systems and evaluation methods for structural performance assessment. Surface-mounted strain sensors are often reinstalled on the outer surface of the containment structure, as shown in Figure 1, to collect data for inverse analysis of internal stress and deformation patterns. In this way, monitoring data can be provided from different angles for structural safety assessment, and, by comparing and accurately determining the deformation law inside the nuclear power plant and determining the cause and location of the accident, it is easier for the operator to identify and solve the problem and provide an alternative method for addressing the failure of the internal strain sensors in the structure [11,12]. However, further verification is still required regarding whether the installation of surface-mounted strain sensors is effective for exploring the laws of internal and external deformation.

Furthermore, establishing a numerical simulation model of the containment structure under various loading conditions is essential for calculating both internal and external deformations [13,14]. The CPR1000 containment structure is made of typical prestressed reinforced concrete featuring an internal steel liner. To simulate the mechanics of this structure; deal with any complicated geometry, material, and prestressed cables; and determine the nonlinear behaviors, the Finite Element (FE) method is a typically versatile-in-nature and well-established computing method in civil engineering practice. Lei Zhou et al. [15] built a full-sized detailed three-dimensional nonlinear finite element model using ABAQUS to evaluate the reliability of the containment structure of a nuclear power plant under accident internal pressure. Yi Ping et al. [16] used ANSYS to model the prestressed concrete containment vessel of the CPR1000, analyzing its aseismic ability under combined seismic loads and internal pressure. Jinke Li et al. [17] built a finite element model of the prestressed concrete containment vessel of a nuclear power plant to simulate the process of integrated leakage rate testing. The above studies confirm that FEM numerical simulation is very useful in containment structure monitoring. Therefore, to ensure the reliability and validity of the alternative solution for surface-mounted strain sensors, numerical simulations should be conducted to verify whether the monitoring data obtained from the alternative system can reasonably reflect the deformation patterns of containment structures. Simultaneously, it can provide a theoretical foundation for the implementation of this solution.

To address these challenges, it is essential to ensure high accuracy in the containment structure’s finite element model and rational selection of material parameters [12,18,19]. This study aims to establish a 3D refined numerical model of the containment structure of a CPR1000 nuclear power unit using ANSYS for response analysis under internal pressure. First, the procedure related to the modeling process of the refined FE model for the containment structure is systematically described, which constitutes the kernel of the analysis technique. Subsequently, a numerical simulation of CTT is performed based on the monitoring data of embedded strain sensors, allowing for modifications to be made to the material parameters through trial calculations, fitting procedures, parameter adjustments, and statistical analyses. Finally, the internal and external deformation behaviors of the containment structure of CPR1000 under internal pressure are studied to illustrate the reasonable validity of the proposed technique. This study opens new perspectives for the safe operational evaluation and maintenance of nuclear containment during service.

## 2. Establishment of 3D Refined Model of Containment Based on ANSYS

In this section, the refined FE modeling process for the containment structure of CPR1000 is systematically described, including the simulation of reinforced concrete, the interaction between concrete and prestressed cables, and prestressing force and the steel liner.

### 2.1. Containment Structure of CPR1000

The CPR1000 reactor type represents a second-generation pressurized water reactor nuclear power technology that is predominantly utilized in China at present. The containment structure of CPR1000 features a complex geometric configuration. To ensure the reliability and durability of this structure, it is crucial to perform detailed modeling and analysis during both the design and operational phases [20,21,22,23].

**Figure 2:**
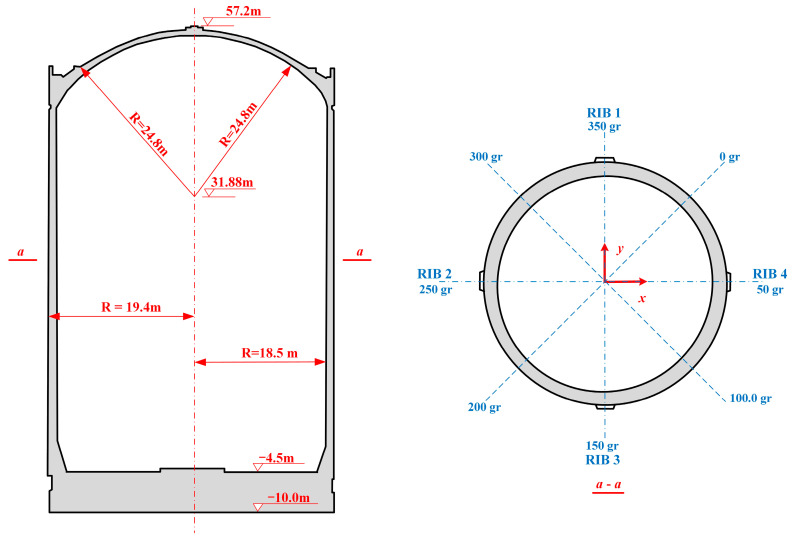
The vertical and horizonal sections of CPR1000 containment.

The containment structure is a typical example of a prestressed reinforced concrete design, characterized by its closely sealed configuration, which comprises a prestressed reinforced concrete wall, dome, and thick base slab, as illustrated in Figure 2. The containment features a single-layer prestressed reinforced concrete shell designed to withstand a maximum internal pressure of 0.42 MPa and an operational temperature of 145 °C.

The containment which is the third barrier of the NPP is cylindrical in shape, with an elliptical dome at the top. The inner diameter of the containment measures 37.0 m, with a thickness of 0.9 m, and the outer diameter is 38.8 m. Additionally, the dome has a thickness of 0.8 m and reaches a total height of 12.37 m, extending from +44.83 to +57.20 m in elevation. The wall ring shell varies from −4.50 to +44.83 m in elevation and is 49.33 m in height. The base slab varies from −10.00 to −4.50 m in elevation and is 5.5 m in thickness.

The inner surface of the containment is lined with fully sealed carbon steel that is 6 mm in thickness and reinforced with vertical and horizontal stiffeners. The steel liner is equipped with 167 distinct sizes of penetrating components, in addition to numerous polar crane brackets and related elements. The largest opening is the equipment hatch, which features a central elevation of +22.90 m and a diameter of 7.4 m. The second opening is the emergency air lock and the personnel air lock, with a diameter of 2.9 m, and their central elevations are +1.15 and +9.15 m, respectively. The height of the internal space is 59.28 m, the total internal volume is about 60,000 m^3^, and the free space volume is about 49,400 m^3^. The containment is sized to ensure sufficient free space to accommodate a hypothetical main loop water loss incident and the need to place a ring crane to load and unload the required large process equipment. Furthermore, anchor nails are uniformly distributed across the concrete base slab, wall, and dome structures to ensure stability, and a fixed steel frame structure is positioned at the base to effectively secure the steel liner flooring.

Four ribs that are 90° from each other are arranged on the outside of the containment wall, as illustrated in the horizonal section in Figure 2. In this study, the coordinate system is defined as follows: the *x*-axis is the center line of ribs 2 and 4, and the positive direction points to rib 4; the *y*-axis is the center line of ribs 1 and 3, and the positive direction points to rib 1; and the positive direction of the z-axis is vertically upward, which is the elevation coordinate axis.

### 2.2. Three-Dimensional Refined FE Model

**Figure 3:**
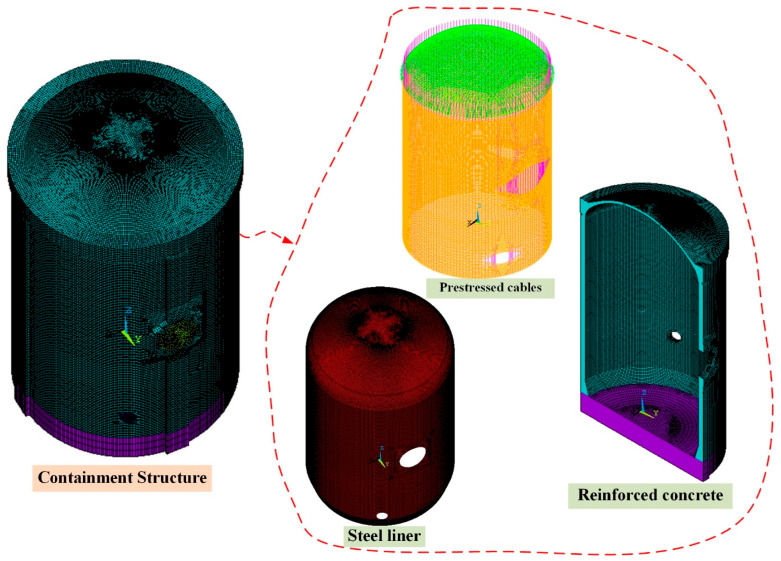
The 3D refined FE model of CPR1000 containment.

The 3D refined FE model of the CPR1000 containment which is established based on ANSYS [24] and used for strain monitoring analysis is shown in Figure 3. According to the design drawings, the reinforced concrete wall and base slab is modeled by the 8-node solid element (SOLID65 in ANSYS), the prestressed cables are modeled by the 2-node bar element (LINK180 in ANSYS), and the steel liner is modeled by the 4-node shell element (SHELL181 in ANSYS). In this study, only static analysis is considered, so the bottom boundary conditions of the raft foundation are fixed constraints. In addition, to better compare and analyze the monitoring data of the strain sensor, the mesh density is an important parameter in the refinement calculation, so the maximum length of the model’s elements does not exceed 350 mm. As a result, this 3D refined FE model of containment is modeled with 606,373 elements and 685,229 nodes in all.

The materials of the containment structure mainly include concrete, rebar, steel plates, and cables. Based on the studies presented in [25,26,27], the material of concrete and tendons was still in the elastic stage when the inner pressure was under 5.85 bar.g. Since this study primarily focuses on the behavior of the containment vessel within the internal pressure range of 0 to 4.2 bar.g, the constitutive relationship adopted in the finite element model is linear elastic. As the containment structure approaches completion, its material parameters are generally at the design values, which are shown in Table 1.

#### 2.2.1. Simulation of Reinforced Concrete

**Figure 4:**
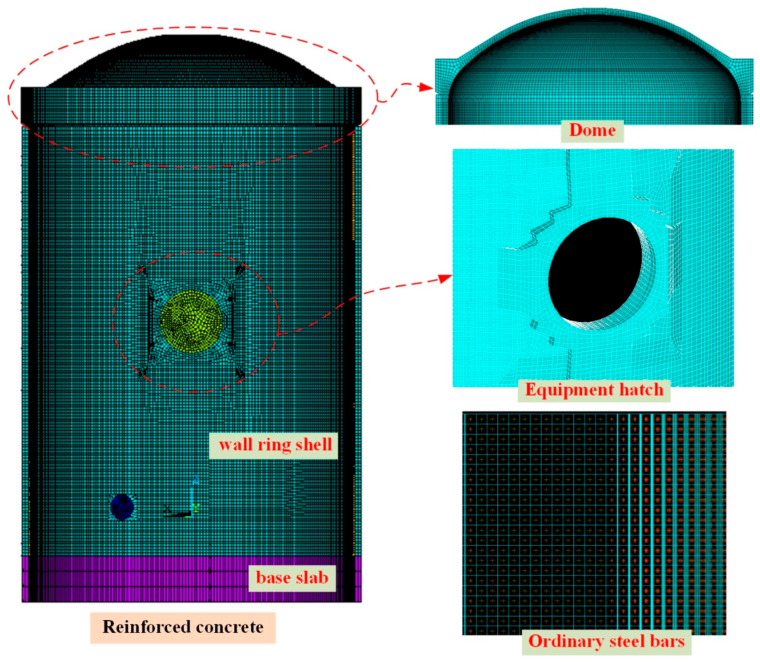
The finite element model of reinforced concrete parts of the containment structure.

The reinforced concrete parts of the containment structure, including the wall ring shell, dome, ribs, equipment hatch, and base slab, are modeled by SOLID65. Due to the structural complexity at the equipment hatch, a limited number of 6-node pentahedron solid elements are also employed as supplementary components. The wall ring shell is divided into six layers along the thickness direction, while the dome is segmented into five layers. Figure 4 illustrates the finite element model of the reinforced concrete parts of the containment structure.

The influence of ordinary steel bars is primarily addressed through both discrete and smeared models in reinforced concrete structures using ANSYS. The discrete model simulates steel reinforcement using bar elements or tube elements, along with interface treatment involving concrete solid elements. While this model yields calculation results that more accurately reflect actual conditions by accounting for the bond and slip between the steel bars and concrete, it presents challenges such as non-convergence during calculations and increased modeling complexity. Conversely, the smeared model assumes a rigid connection between ordinary steel bars and concrete, allowing for the configuration of three types of steel bars oriented in different directions by adjusting parameters within SOLID65. This model offers convenience in modeling and tends to converge more readily. Given the presence of numerous structural members, complex layouts of reinforcing bars, and the need to consider the effects of prestressed cables, the integral model is adopted for structural analysis. The volume reinforcement ratio of the containment structure is presented in Table 2, and the rebar orientations of the partial wall are illustrated in Figure 4.

#### 2.2.2. Interaction Between Concrete and Prestressed Cables

The prestressed cables and the concrete may experience slip under external loads, necessitating consideration of contact. However, the anchorage between these components is relatively secure in nuclear containment structures due to the post-tensioned construction. Even if slip occurs, the resulting gaps are minimal. Furthermore, the CTT load for the containment structure falls within the design load range. Therefore, the potential contact effects between cables and concrete are neglected. Additionally, the concrete and prestressed cables are modeled separately, and then the interaction between concrete and prestressed cables is realized by establishing the constraint equation (ceintf command) between the nodes of concrete and cables.

**Figure 5:**
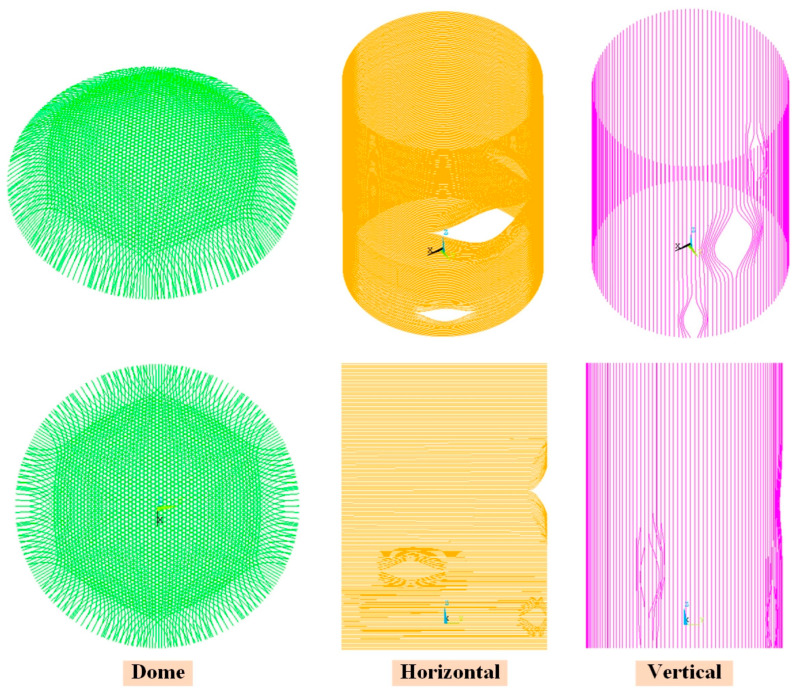
The LINK180 elements for prestressed cables.

The prestressed cables are modeled using the two-node three-dimensional bar element LINK180, which is then integrated into the concrete model of the containment structure, and the finite element representation of the prestressed cables is illustrated in Figure 5. In accordance with the design specifications, the dome cables consist of 3 sets defined by direction (rotation by 2π/3), each on the revolution tracing surfaces. There are 174 dome cables in total, with different lengths from 26 to 44 m, and the pre-tension force applied by each cable is 430 t, for a total of 74,820 t. Two types of vertical and horizontal prestressed cables are installed on the ring shell structure. There are 144 vertical cables from the top elevation of −12.50 m on the top of the prestressed corridor to +51 m, and the distance between the two adjacent axes is 820 mm. Each cable can apply 810 t of prestress, for a total of 117,000 t. The horizontal prestressed cables are placed in the range of the ring shell elevation from −4.5 to +45.60 m; the axis distance between the two upper and lower adjacent sleeves is 220 mm; the two inner and outer layers of prestressed cables are set, both in the inner side of the vertical prestressed steel bundle; and the axis distance between the two inner and outer layers is 200 mm. A total of 223 cables are adopted in the full circumference, and the pre-tension force applied by each cable is 430 t, for a total of 95,900 t. All the prestressed cables are pressurized into the casing to prevent air corrosion after the prestressed tension is completed. In addition, the cross-sectional areas of the LINK180 elements of the dome and vertical and horizontal prestressed cables in the FE model are 2850, 5400, and 2850 mm^2^, respectively.

The application of pre-tension can be effectively simulated using a cooling method. This method involves applying a temperature drop value, Δ*T*, to the elements of prestressed cables. Such an application simulates the effect of prestressed tension resulting from the shrinkage of force cables due to temperature changes. The relationship governing the temperature drop is expressed by the following formula:

Δ*T* = *σ*/(*Eα*) (1)

where *σ* represents the applied value of pre-tension force, *E* denotes the elastic modulus of prestressed cables, and *α* signifies the linear expansion coefficient of these cables.

#### 2.2.3. Simulations of Steel Liner, Penetration, and Hatches

The steel liner covering the inner surface of the containment structure is equipped with reinforcement ribs in vertical and horizontal directions, and anchor nails are uniformly distributed across the concrete base slab, wall, and dome to ensure stability. Therefore, the steel liner, penetration, and hatches are modeled by the 4-node *shell element* and the interaction between concrete and the steel liner is realized by defining coupled degrees of freedom at the position of anchor nails (CPINTF command).

**Figure 6:**
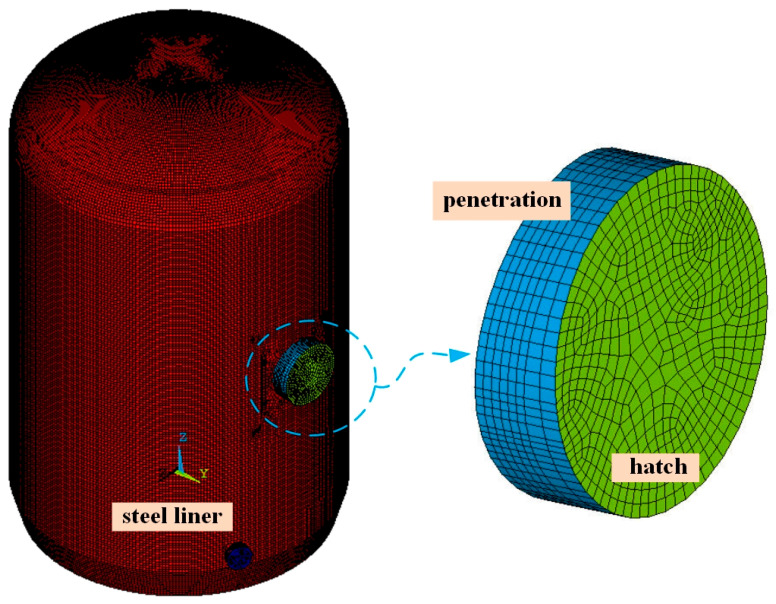
The SHELL181 elements for the steel liner, penetration, and hatches.

As illustrated in Figure 6, each element of the steel liner, penetration, and hatches typically measures 300 mm × 300 mm, while the thickness of each shell element of the steel liner is 6 mm. In addition, three openings with a diameter greater than 2 m on the wall ring shell were considered in the modeling process. The penetration and equipment hatch are represented by shell elements with thicknesses of 16 and 300 mm, respectively. The penetrations of the emergency air lock and the personnel air lock are modeled using shell elements with thicknesses of 20 and 100 mm, respectively.

## 3. Strain Monitoring During CTT

As previously mentioned, the EAU system serves as a health feedback mechanism to monitor the operational status of the containment structure and ensure the safe operation of the reactor. Surface-mounted strain sensors are strategically placed on the surface of the containment structure in correspondence with embedded strain sensors; this allows for data measurement and comparison during CTT phases. On one hand, measured data can be provided for the numerical calculation model; on the other, it can demonstrate the feasibility of using the surface-mounted strain sensors instead of evaluating the structural mechanical performance when the embedded strain sensors of the containment are abnormal.

To ensure the accuracy and timeliness of the data, an automatic data acquisition system is employed for monitoring purposes. During the acquisition process, manual intervention is unnecessary, and the data is automatically collected and stored by the computer at 15 min intervals. The entire system comprises a computer and a data acquisition setup DATATAKER, which includes the host YJDT-85G and the CEM20 data acquisition module.

### 3.1. Layout of Strain Sensors

**Figure 7:**
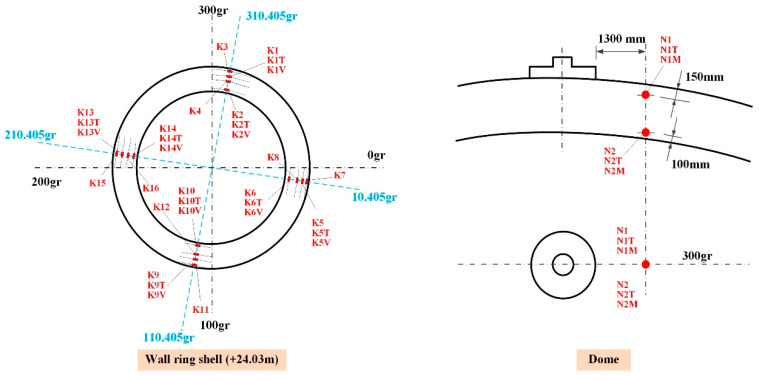
Location of strain sensors of containment and dome.

The embedded strain sensor is installed in four generatrixes at the wall ring shell, the two inside and outside layers, each in three directions (vertical **V**, radial **R,** and toroidal **T**), as well as in two layers and two orientations (Toroidal **T** and Meridional **M**) at the dome. As shown in Figure 7, the original measuring points are all distributed at an elevation of +24.03 m, from inside to outside: K2/K4/K1/K3 at 310 gr, K6/K8/K5/K7 at 10 gr, K10/K12/K9/K11 at 110 gr, and K14/K16/K13/K15 at 210 gr. The newly installed surface-mounted strain sensors are positioned on the concrete surface in alignment with the original measurement points.

### 3.2. Load Condition During CTT

The containment structure is subjected to various loads during the CTT, including structural self-weight, temperature load, equipment load, and internal pressure load. Among these, the structural self-weight and equipment load are considered constant loads acting on the containment. The containment structure was basically in a stable state under the constant load mentioned above before conducting the CTT. Consequently, the influence on the numerical variation of strain sensors is minimal and can often be disregarded. In subsequent numerical calculations, the primary load that induces changes in strain sensor readings is internal pressure.

**Figure 8:**
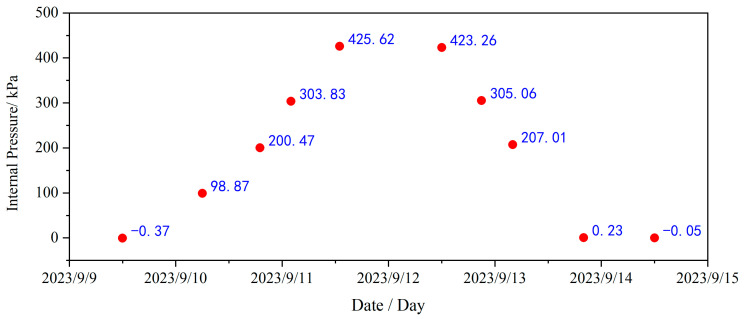
Internal pressure during CTT.

Referring to the relevant test data from CTT, the typical internal pressure values of the containment from 10 September 2023 to 17 September 2023, along with their variation trends during this period, are illustrated in Figure 8. It can be seen that the internal pressure values predominantly range between about 0.0 and 4.2 bar.g, with an overall linear trend observed across each interval.

### 3.3. Monitoring Data Analysis

Although the containment structure has undergone deformation due to self-weight and pre-tension loads, it remains fundamentally stable under constant-load conditions. In this stage, the strain sensor has not yet commenced monitoring. Consequently, the strain of concrete measured by the strain sensor is utilized to assess the performance of the containment during CTT.

**Figure 9:**
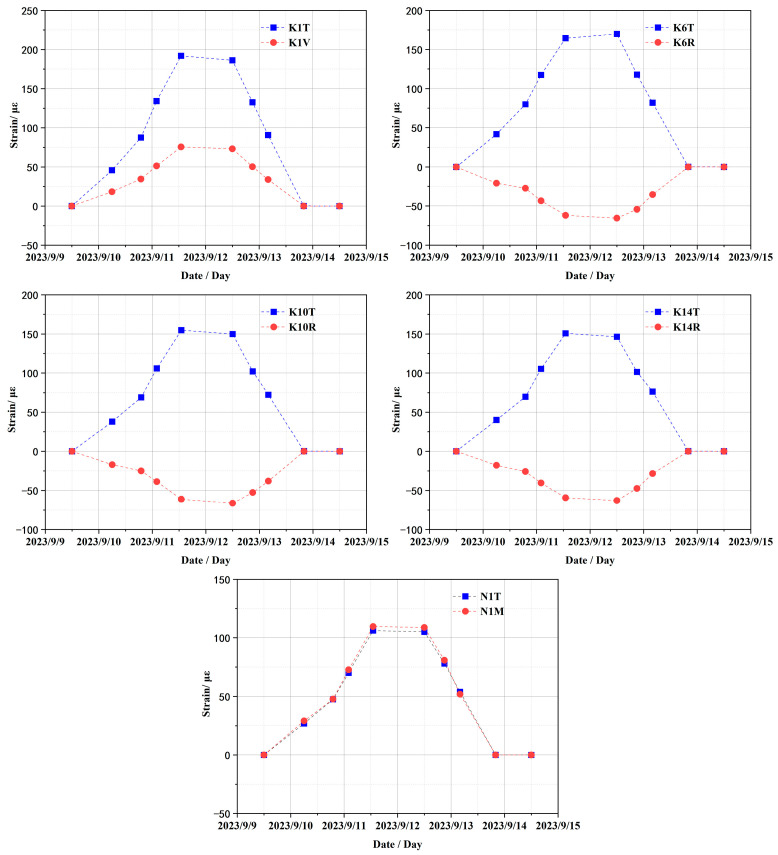
Variation curves of strain monitoring data of typical sensors.

The variation curves of strain monitoring data from embedded sensors at typical measurement points during the CTT are presented in Figure 9, where strain sensor IDs (K1, K6, K10, K14, N1) correspond to locations in Figure 7 with the following directional suffixes: “T” = toroidal strain, “V” = vertical strain, “R” = radial strain, and “M” = meridional strain. The variation curves of the figure reveal that the monitoring data from all strain sensors can effectively reflect the clear pressure platform and basically return to the strain state before pressure application after pressure relief. In subsequent comparative analyses between test data and simulation results, adjustments will be made to the finite element calculation model based on the aforementioned strain data.

## 4. Numerical Simulation Analysis

As a prestressed concrete structure, the material composition of the containment structure is highly complex. Although construction is conducted in accordance with the design drawings, variations in material selection, concrete mix design, fabrication methods, processing techniques, and construction processes can lead to discrepancies. Furthermore, the material will deteriorate with service time, and the parameters such as elastic modulus, Poisson’s ratio, material properties, and prestressing forces of cables exhibit randomness. Consequently, sensitivity analysis of the material parameters is needed to modify the finite element calculation model to obtain the structural response of the strain, stress, and displacement of the entire containment under different working conditions and predict the structural response of the containment under different service times in the future. This section employs the 3D refined FE model established in Section 2.2 to simulate CTT during overhauls and perform sensitivity analysis on these parameters. By comparing and analyzing the numerical results of the containment under internal pressure with strain measurement data obtained from embedded strain sensors, adjustments are made to the initial structural parameters of the model to ensure reasonable reliability of the calculated data.

### 4.1. Sensitivity Analysis of Material Parameters

To investigate the sensitivity and variation patterns of strain responses in the containment structure under different material parameters, the elastic modulus and Poisson’s ratio of concrete and cables combined with the inspection data are considered. The detailed work case of the sensitivity analysis for the calculation is shown in Table 3. The other parameters are kept the same as the initial designed values. For comparative clarity, the strain sensor monitoring values herein use notations such as “K1T” or “K2V” for the wall ring shell, where K1-K16 denote sensor IDs in Figure 7 with suffixes “V” (vertical strain), “R” (radial strain), and “T” (toroidal strain), while dome strain sensors use “N1T” notations, where N1-N2 indicate sensor IDs with suffixes “T” (toroidal strain) and “M” (meridional strain). Numerical results are denoted by a hyphen (“-”) followed by the Work Case ID specified in Table 3. For example, “K1V-E1” represents the vertical strain at the K1 sensor location when the concrete elastic modulus is 35.0 GPa.

#### 4.1.1. Elastic Modulus of Concrete

**Figure 10:**
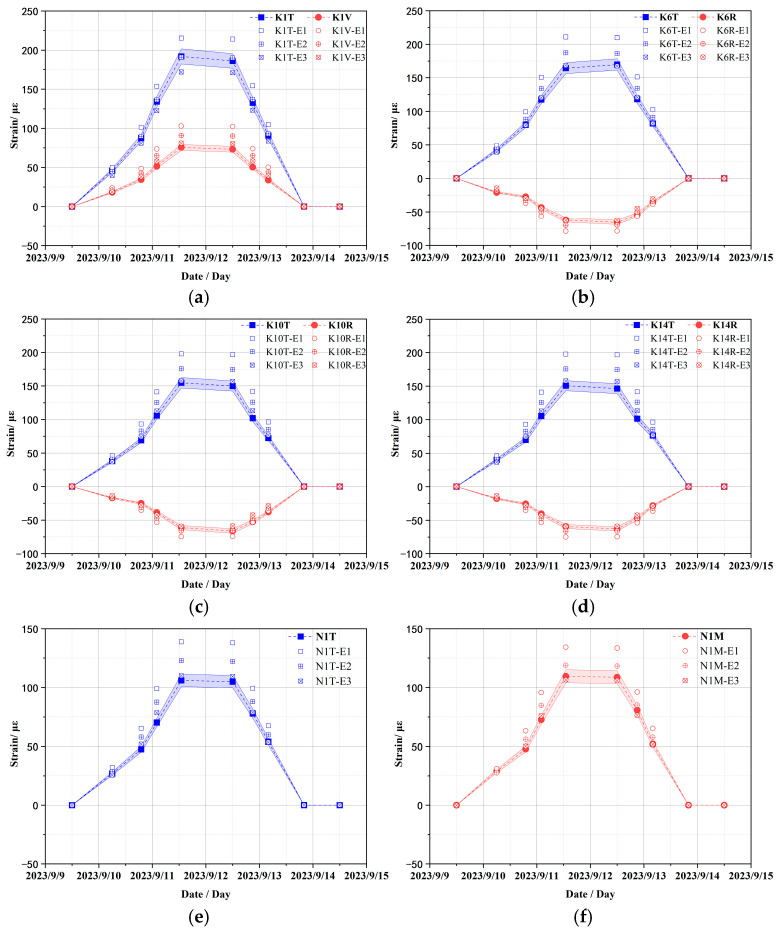
Comparison of strain results under different elastic moduli of concrete (E1 = 35.0 GPa; E2 = 40.0 GPa; E3 = 45.0 GPa). (**a**) Vertical and toroidal strains at K1 sensor location; toroidal and radial strains at (**b**)–(**d**) K6, K10, and K14 sensor locations, respectively; (**e**) toroidal strain at N1 sensor location; (**f**) meridional strain at N1 sensor location.

Based on the monitoring strain data obtained during CTT, the comparison curves between the numerically calculated strain data and the monitoring data under different concrete elastic moduli of concrete conditions are shown in Figure 10. For the convenience of comparative analysis, the error band is restricted to 5% of the monitoring strain data value at each monitoring time point.

What is evident from the figure is that the elasticity modulus of concrete significantly influences the strain of the containment structure. Specifically, it has been observed that as the elastic modulus increases, the absolute value of strain decreases. When comparing these findings with monitoring data, it becomes clear that the calculated strain values using an elastic modulus of 45.0 GPa align well with most of the measured results and the errors are also less than the acceptable 5% error margin; particularly, the clear pressure platform is well reflected and basically returns to the strain state before pressure application after pressure relief. Under this condition, the calculated strain values of K1T are only marginally less accurate than those obtained under two other conditions at the peak measured pressure; however, the overall change trend is well consistent, and the amplitude difference is not large. This discrepancy can primarily be attributed to the positioning of the K1 strain sensor near the equipment hatch (elevation +24.0 m; diameter 7.4 m), and the surrounding mesh configuration is irregular, which leads to differences between the layout of prestressed cables and conventional reinforcement bars compared to actual conditions, thereby affecting numerical calculation outcomes for strain values to some extent. The above results show that the elastic modulus of concrete plays a crucial role in determining strain values of the containment structure. The calculated strains corresponding to an elastic modulus of 45.0 GPa demonstrate strong concordance with most monitoring data and may serve as reliable parameters for subsequent FE model numerical calculations.

#### 4.1.2. Poisson’s Ratio of Concrete

**Figure 11:**
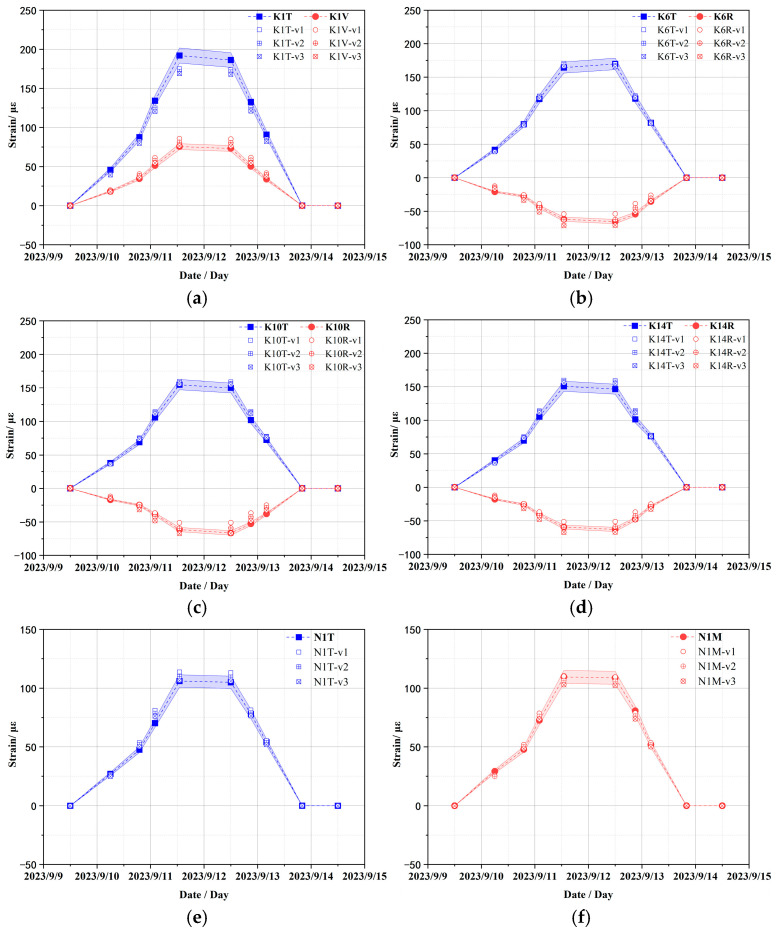
Comparison of strain results under different Poisson ratios of concrete (v1 = 0.17; v2 = 0.20; v3 = 0.23). (**a**) Vertical and toroidal strains at K1 sensor location; toroidal and radial strains at (**b**–**d**) K6, K10, and K14 sensor locations, respectively; (**e**) toroidal strain at N1 sensor location; (**f**) meridional strain at N1 sensor location.

The strain values at typical measuring points of the containment structure were analyzed under different Poisson ratios of concrete, as shown in Figure 11. Overall, the change in Poisson’s ratio of concrete has a slight influence on the strain data of the wall ring shell and dome of the containment structure with a concrete elastic modulus of 45.0 GPa. Importantly, when the internal pressure is large, the toroidal and vertical strains decrease slightly with the increase in Poisson’s ratio. Therefore, the initial value of 0.2 is still selected for the Poisson ratio of concrete in the subsequent numerical calculation.

#### 4.1.3. Material Parameters of Prestressed Cables

**Figure 12:**
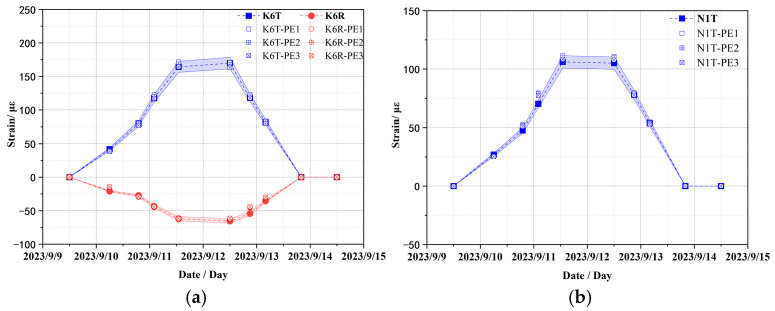
Comparison of strain results under different elastic moduli of prestressed cables (PE1 = 190.0/1.5 GPa; PE2 = 190.0 GPa; PE3 = 190.0 × 1.5 GPa). (**a**) Toroidal and radial strains at K6 sensor location; (**b**) toroidal strain at N1 sensor location.

**Figure 13:**
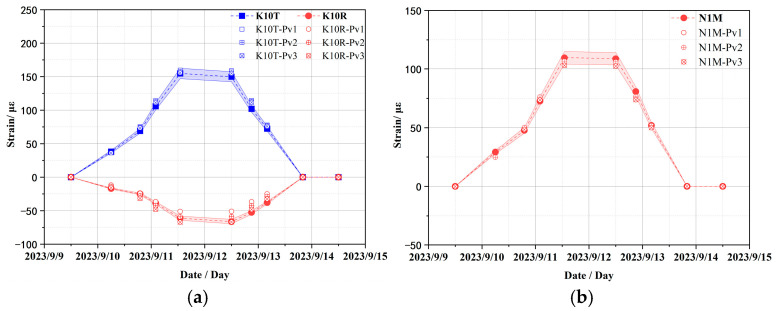
Comparison of strain results under different Poisson ratios of prestressed cables (Pv1 = 0.25; Pv2 = 0320; Pv3 = 0.35). (**a**) Toroidal and radial strains at K10 sensor location; (**b**) meridional strain at N1 sensor location.

In addition, the sensitivity analysis of material parameters of prestressed cables as shown in Table 3 was also carried out to explore the influence on strain response. Owing to spatial confinement, this study enumerates typical strain results only. Figure 12 compares the strain responses of the containment structure at strain sensors K6 and N1 under different elastic moduli of the prestressed cables, while Figure 13 compares those at strain sensors K10 and N1 under different Poisson ratios of the prestressed cables. The overall variant trend of the structure is similar across all work cases and the errors are also mostly less than the acceptable 5% error margin, with only slight differences in strain amplitudes. In other words, the elastic modulus and Poisson ratio of prestressed cables have a slight effect on the strain response of the containment structure. Therefore, the initial value is still selected for the elastic modulus and Poisson ratio of prestressed cables in the subsequent numerical calculation.

### 4.2. Response Analysis of Containment Under Internal Pressure

To comprehensively explore the variation law between the internal and external deformation of the containment structure of CPR1000, a response analysis was conducted under typical CTT loads using the modified model, and the results of the displacement, strain, and stress were obtained.

#### 4.2.1. Response Analysis Under Self-Weight and Prestressed Load

**Figure 14:**
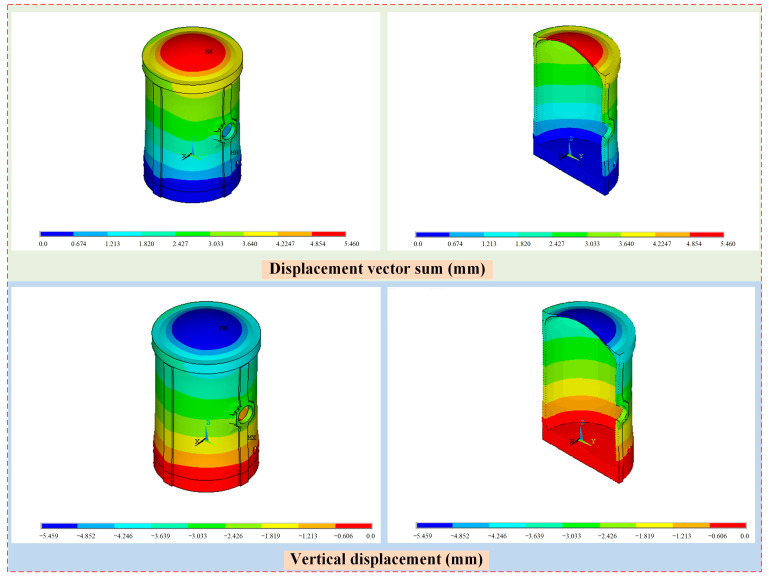
The displacement distribution of the containment structure under self-weight and prestressed load.

The displacement distribution of the containment structure under self-weight and prestressed load is illustrated in Figure 14. From the point of view of displacement distribution, the distribution is generally stratified, and as the elevation increases, the displacement amplitude also increases. From the point of view of the displacement sum, the maximum total displacement is 5.46 mm, which occurs in the dome region. For the vertical displacement of the containment structure, the displacement distribution shows a similar pattern which is also stratified, and the maximum vertical displacement is −5.458 mm, which is the main deformation of the containment structure under self-weight and prestressed load.

**Figure 15:**
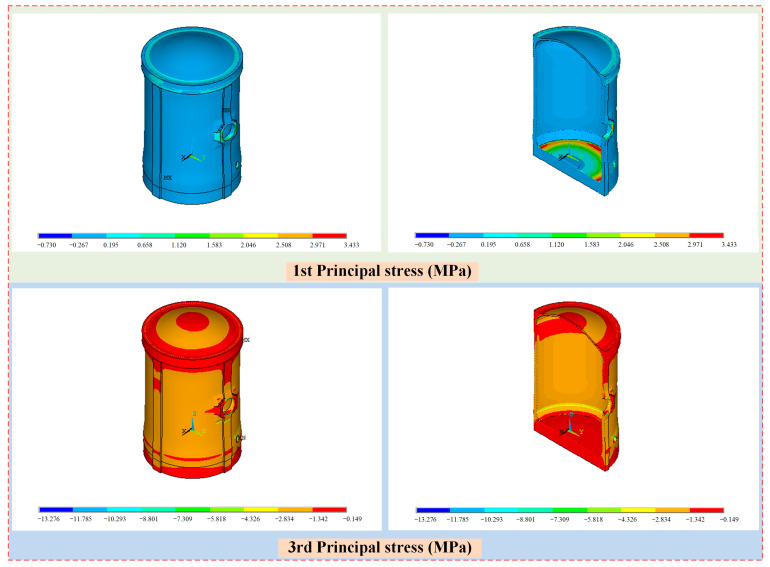
The stress distribution of the containment structure under self-weight and prestressed load.

The contour of maximum principal stresses of the containment structure under self-weight and prestressed load is illustrated in Figure 15. From the point of view of first principal stress distribution, tensile stress was concentrated at the end position of the prestressed cables, the edge of the equipment hatch, and the junction between the frustrum and base slab, while most other areas were under pressure. From the point of view of the stress value, the first principal stress of most positions of the structure was less than about 0.0 MPa, and the stress values of the edge of the equipment hatch and the junction between the frustrum and base slab were larger. These positions indicate the weak regions of the structure that need to be reinforced. In addition, the maximum first principal stress was 3.43 MPa, which occurred at the junction between the frustrum and base slab.

For the third principal stress of the containment structure, the wall ring shell is in a slightly pressurized state, which is mainly due to the role of prestressed cales, and the stress of the dome part and base slab is less than that of the wall on the whole. From the point of view of stress values, the third principal stress of most positions of the structure is about −2.0 MPa, and the stress values at the edge of the hatch and the junction between the frustrum and base slab are larger. In addition, the minimum third principal stress is −13.3 MPa, which occurs at the edge of the emergency air lock, which is less than the compressive strength of concrete.

**Figure 16:**
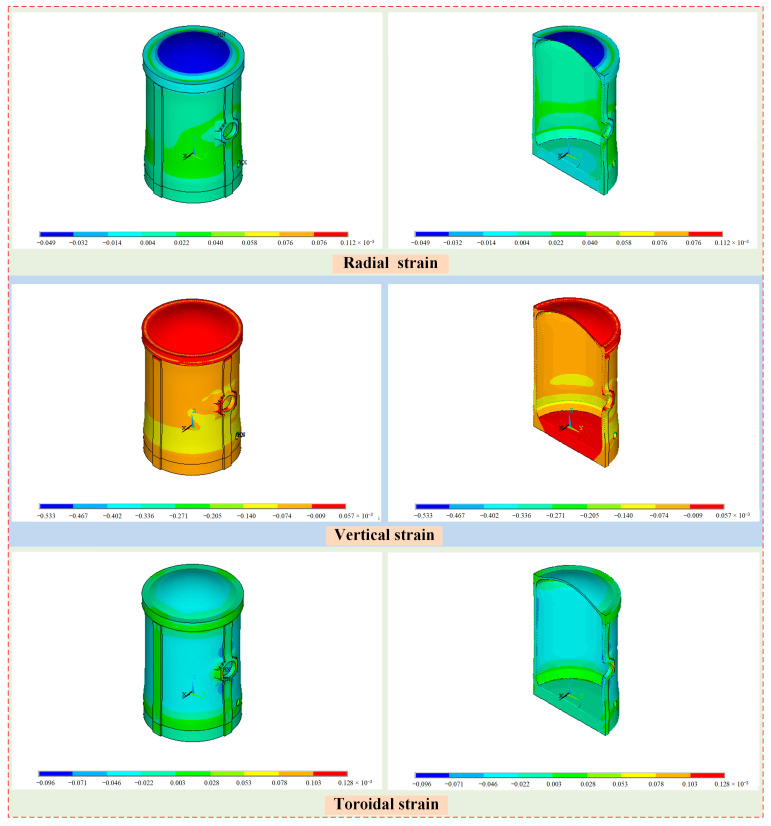
The strain distribution of the containment structure under self-weight and prestressed load.

The strain distribution of the containment structure under self-weight and prestressed load is shown in Figure 16. For the radial strain of the structure, the strain values are small in the dome region, while the values are large at the edges at openings such as the hatches. The main distribution range is [−0.049 × 10^−3^, 0.112 × 10^−3^], and the maximum radial strain occurs at the edge of the emergency air lock. For the vertical strain of the containment structure, the strain of the whole wall ring shell is large, especially in the lower part of the wall and the emergency air lock, while the values are small in the dome and base slab. The main distribution range is [−0.532 × 10^−3^, 0.057 × 10^−3^], and the minimum vertical strain occurs at the edge of the emergency air lock. For the toroidal strain of the containment structure, the strain at the end position of the prestressed cables, frustrum, and the ribs are slightly large, especially in the equipment hatch, while the values are small in the wall ring shell except the ribs. The main distribution range is [−0.096 × 10^−3^, 0.128 × 10^−3^], and the maximum toroidal strain occurs at the edge of the equipment hatch.

#### 4.2.2. Response Analysis Under Internal Pressure

**Figure 17:**
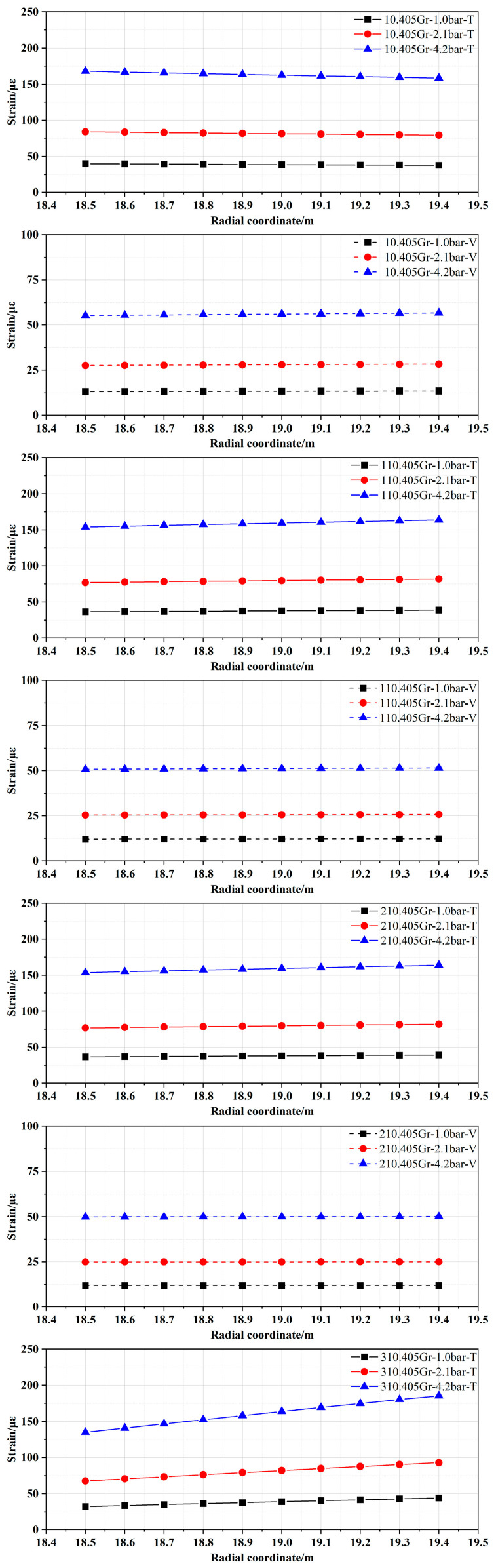

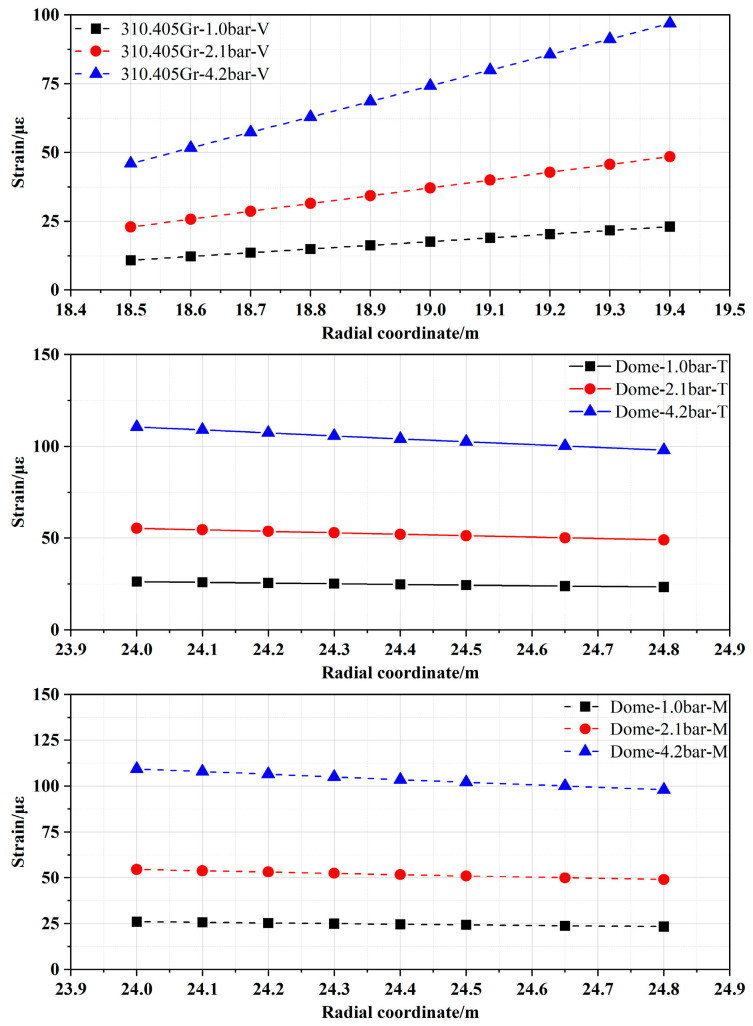
The strain variation curves at typical positions under different internal pressure values.

Based on the modified FE model of the containment structure, a further investigation into the variation laws of both internal and external strain responses was carried out under internal pressure. In order to facilitate a comparative analysis, the strain variation curves along the thickness of the containment structure at selected typical points located at an elevation of 24.0 m and at the dome (the location of embedded strain sensors as shown in Figure 7) under three typical internal pressure values (1.0, 2.1, and 4.2 bar.g) are illustrated in Figure 17, and the notation in the figure is the same as before. For example, the notation “10.405 Gr-1.0 bar-T” in the figure indicates the toroidal strain at the 10.405 Gr location of the wall ring shell under 1.0 bar.g of internal pressure.

From the results presented in Figure 17, with the increase in the internal pressure value, the overall strain at each monitoring point increases accordingly. However, the strain at different monitoring points also shows different variations. For the two locations—110.405 and 210.405 Gr—which are situated far from the equipment hatch, the variation patterns of toroidal and vertical strains exhibit a gradual increase from the inner to the outer surface along the thickness direction of the wall under three typical internal pressure values, and the magnitude of change remains relatively small. For example, the vertical strain of the innermost side of the wall at 110.405 Gr is 12.10 με under the internal pressure value of 1.0 bar.g, while the strain of the outermost side of the wall is 12.26 με. By contrast, for the two locations—10.405 and 310.405 Gr—which are situated near the equipment hatch, strain variations are significantly influenced by the spatial curvature arrangement of prestressed cables. Although there is a gradual increase in strain from inside to outside along the thickness direction as well, the strain values at 310.405 Gr experience a larger amplitude change; especially, the larger value of internal pressure is clearer. For example, the vertical strain of the innermost side of the wall is 45.92 με under an internal pressure of 4.2 bar.g, while the strain of the outermost side of the wall is 96.95 με. However, the toroidal strain at 10.405 Gr has a gradual decreasing trend from the inner to the outer side along the thickness of the wall, although the variation amplitude is small. The vertical strain shows similar behavior to that observed at locations 110 and 210 Gr. For the dome, both toroidal strain and meridional strain exhibit a gradual decrease in the thickness direction, transitioning from the inner wall to the outer side. Furthermore, it can be observed that the amplitude of strain variation on both the inner and outer sides remains relatively small across different internal pressure conditions. In addition, the trends in strain under internal pressures of 2.1 and 4.2 bar.g mirror those observed under an internal pressure of 1.0 bar.g, but the strain and the amplitude of strain variation between the inner and outer sides will increase with the increase in the internal pressure.

## 5. Conclusions

A three-dimensional refined numerical simulation model of the containment structure of a CPR1000 nuclear power unit is established using ANSYS for response analysis during CTT, which includes simulations of prestressed cables, the application of pre-tension, ordinary steel bars, and the interactions between the steel liner and concrete. Then, a numerical simulation of CTT is performed based on the monitoring data of embedded strain sensors to modify the material parameters of the model, and the elastic modulus of concrete has a greater influence. Meanwhile, the results of the refined modeling analysis can be highly consistent with the monitoring strain of CTT, and the errors are also mostly less than the acceptable 5% error margin. Finally, the containment structure of a CPR1000 subjected to internal pressure excitation was comprehensively evaluated from the discussion of structural stress, deformation, and strain, and the variation law between the internal and external deformation of the containment structure was also revealed. The following conclusions were drawn:

(1) The displacement distribution of the containment structure is generally stratified, and as the elevation increases, the displacement amplitude also increases. The maximum total displacement occurs in the dome region under structural self-weight and prestressed load. The principal tensile stress of the edge of the equipment hatch and the junction between the frustrum and base slab is larger, while the principal compressive stress meets the strength requirements of concrete. The larger strain values mainly appear at the end position of the prestressed cables, frustrum, the ribs, and the edge of the equipment hatch. These positions are the weak region of the structure and need to be maintained.
(2) The spatial curvature arrangement of prestressed cables near the equipment hatch significantly influences strain variations. The strain values of the wall ring shell, located far from the equipment hatch, exhibit a linear increase with a relatively small amplitude of change. Although there is a gradual increase in strain from inside to outside along the thickness direction as well, the strain values at 310.405 Gr experience a larger amplitude change; especially, the larger value of internal pressure is clearer. The toroidal strain at 10.405 Gr has a gradual decreasing trend from the inner to the outer side along the thickness of the wall, although the variation amplitude is small. The vertical strain shows similar behavior to that observed in other regions.
(3) The strain of the dome exhibits a gradual decrease in the thickness direction and the amplitude of strain variation on both the inner and outer sides remains relatively small across different internal pressure conditions.
(4) Sensor systems and refined FEM simulation are very useful in containment structure monitoring, and the method of adding surface-mounted strain sensors to the surface to invert the internal stress and deformation law is feasible. However, the reliability of monitoring data of instruments such as embedded strain sensors and temperature sensors is the basis of numerical simulation. Meanwhile, due to the difference in local regularity caused by the uneven distribution of prestressed tendons at the equipment hatches, it is recommended to add other surface monitoring sensors to further improve reliability in practical engineering applications.

The refined 3D model developed in this study is applicable to elastic-range simulations under CTT-like pressure testing conditions. However, it cannot capture the nonlinear behavior of concrete or prestressed cables. Future research should extend this model by incorporating nonlinear material properties to assess the structural reliability of nuclear containments under severe accident pressure scenarios.

## Figures and Tables

**Table 1:** The design material parameters of the containment structure.

Parts	Materials	Density (kg/m^3^)	Elasticity Modulus (GPa)	Poisson’s Ratio
Rebar1	HPB400	7850	206	0.3
Rebar2	HRB335	7850	206	0.3
Concrete	C50	2300	40.0	0.2
Hatch	Rolled steel	7850	206	0.3
steel liner	P265GH (325 MPa)	7850	206	0.3
prestressed cables	Low-relaxation prestressed steel strand (1770 MPa)	7850	190	0.3

**Table 2:** The volume reinforcement ratio of the containment structure.

Component	Wall Ring Shell				Dome		
Direction	Vertical		Horizontal		Internal	Central	External
	Internal	External	Internal	External			
Below 30 m	0.20%	0.20%	0.22%	0.20%	0.06%	0.32%	0.20%
30 m–40 m	0.16%	0.20%	0.10%	0.16%			
Above 40 m	0.19%	0.19%	0.16%	0.16%			

**Table 3:** The work case of the sensitivity analysis of material parameters.

Analysis Parameter	Work Case	Elasticity Modulus of Concrete (GPa)	Poisson’s Ratio of Concrete	Elasticity Modulus of Cables (GPa)	Poisson’s Ratio of Cables
Elasticity modulus of concrete	E1	35.0	0.20	190.0	0.30
	E2	40.0	0.20	190.0	0.30
	E3	45.0	0.20	190.0	0.30
Poisson’s ratio of concrete	v1	45.0	0.17	190.0	0.30
	v2	45.0	0.20	190.0	0.30
	v3	45.0	0.23	190.0	0.30
Elasticity modulus of cables	PE1	45.0	0.20	190.0/1.5	0.30
	PE2	45.0	0.20	190.0	0.30
	PE3	45.0	0.20	190.0 × 1.5	0.30
Poisson’s ratio of cables	Pv1	45.0	0.20	190.0	0.25
	Pv2	45.0	0.20	190.0	0.30
	Pv3	45.0	0.20	190.0	0.35

## Data Availability

Data will be made available on request.
